# Estimated oxygen extraction versus dynamic parameters of fluid-responsiveness for perioperative hemodynamic optimization of patients undergoing non-cardiac surgery: a non-inferiority randomized controlled trial

**DOI:** 10.1186/s12871-020-01011-z

**Published:** 2020-04-18

**Authors:** Andrea Carsetti, Mirco Amici, Tonino Bernacconi, Paolo Brancaleoni, Elisabetta Cerutti, Marco Chiarello, Diego Cingolani, Luisanna Cola, Daniela Corsi, Giorgio Forlini, Marina Giampieri, Salvatore Iuorio, Tiziana Principi, Giuseppe Tappatà, Michele Tempesta, Erica Adrario, Abele Donati

**Affiliations:** 1 Department of Biomedical Sciences and Public Health, Università Politecnica delle Marche, Ancona, Italy; 2 Anesthesia and Intensive Care Unit, Azienda Ospedaliero Universitaria Ospedali Riuniti, Ancona, Italy; 3 Pediatric Anesthesia and Intensive Care Unit, Azienda Ospedaliero Universitaria Ospedali Riuniti, Ancona, Italy; 4 Anesthesia and Intensive Care Unit, ASUR Marche, area vasta n. 2, Jesi, Italy; 5 Anesthesia and Intensive Care Unit, ASUR Marche, area vasta n. 1, Urbino, Italy; 6 Anesthesia and Post-operative Intensive Care Unit, Azienda Ospedaliero Universitaria Ospedali Riuniti, Ancona, Italy; 7 Anesthesia and Intensive Care Unit, ASUR Marche, area vasta n. 3, Camerino, Italy; 8 Anesthesia and Intensive Care Unit, ASUR Marche, area vasta n. 2, Senigallia, Italy; 9 Anesthesia and Intensive Care Unit, ASUR Marche, area vasta n. 4, Fermo, Italy; 10 Anesthesia and Intensive Care Unit, ASUR Marche, area vasta n. 3, Civitanova Marche, Italy; 11 Anesthesia and Intensive Care Unit, ASUR Marche, area vasta n. 5, Ascoli Piceno, Italy; 12 Anesthesia and Intensive Care Unit, IRCCS-INRCA, Ancona, Italy; 13 Anesthesia and Intensive Care Unit, IRCCS-INRCA, Osimo, Italy; 14 Anesthesia and Intensive Care Unit, ASUR Marche, area vasta n. 5, San Benedetto del Tronto, Italy; 15 Anesthesia and Intensive Care Unit, ASUR Marche, area vasta n. 3, Macerata, Italy; 16 Anesthesia and Intensive Care Unit, Azienda Ospedaliera Marche Nord, Pesaro, Italy

**Keywords:** Perioperative goal directed therapy, Fluid-responsiveness, Oxygen extraction rate, Complications

## Abstract

**Background:**

Goal directed therapy (GDT) is able to improve mortality and reduce complications in selected high-risk patients undergoing major surgery. The aim of this study is to compare two different strategies of perioperative hemodynamic optimization: one based on optimization of preload using dynamic parameters of fluid-responsiveness and the other one based on estimated oxygen extraction rate (O_2_ER) as target of hemodynamic manipulation.

**Methods:**

This is a multicenter randomized controlled trial. Adult patients undergoing elective major open abdominal surgery will be allocated to receive a protocol based on dynamic parameters of fluid-responsiveness or a protocol based on estimated O_2_ER. The hemodynamic optimization will be continued for 6 h postoperatively. The primary outcome is difference in overall postoperative complications rate between the two protocol groups. Fluids administered, fluid balance, utilization of vasoactive drugs, hospital length of stay and mortality at 28 day will also be assessed.

**Discussion:**

As a predefined target of cardiac output (CO) or oxygen delivery (DO_2_) seems to be not adequate for every patient, a personalized therapy is likely more appropriate. Following this concept, dynamic parameters of fluid-responsiveness allow to titrate fluid administration aiming CO increase but avoiding fluid overload. This approach has the advantage of personalized fluid therapy, but it does not consider if CO is adequate or not. A protocol based on O_2_ER considers this second important aspect. Although positive effects of perioperative GDT have been clearly demonstrated, currently studies comparing different strategies of hemodynamic optimization are lacking.

**Trial registration:**

ClinicalTrials.gov, NCT04053595. Registered on 12/08/2019.

## Background

Any surgical intervention is a trauma for the organism and a stress response is activated to cope the external insult. This stress response is responsible of an increase in oxygen consumption. Shoemaker et al. [1] showed that oxygen debt start intraoperatively in high risk surgical patients. If patient is not able to overcome the deficit in oxygen consumption (VO_2_) during the first hours postoperatively, he/she will go toward complications (in case of delay to meet metabolic demand) or death (in case of persistent VO_2_ deficit). The same authors showed that the incidence of organ failure and mortality were reduced when oxygen debt was rapidly compensated using a protocol of hemodynamic optimization aiming to reach the same hemodynamic targets recorded in survived patients [1]. Therefore, several protocols have been developed to optimize hemodynamic parameters with the aim to reduce tissue hypoperfusion coming from maldistribution or inadequate perfusion and meet the increased metabolic need as soon as possible.

Every patient that probably will not be able to face the surgical stress himself might benefit from modulation of hemodynamic parameters. Actually, goal directed therapy (GDT) is able to improve survival only in high-risk surgical patients [2]. On the other hand, the reduction of complications rate has been shown also in intermediate-risk population [3–7].

Originally, hemodynamic optimization protocols were developed to reach supranormal value for cardiac output (CO) and oxygen delivery (DO_2_) [1]. DO_2_ is locally regulated according to tissue metabolism and an adequate global DO_2_ may coexist with local hypoperfusion. An inadequate regional DO_2_ is responsible for an increase of oxygen extraction, a reduction of mixed venous oxygen saturation (SvO_2_) and central venous oxygen saturation (ScvO_2_) as its surrogate and finally an increase of lactate. Based on the concept that oxygen extraction rate (O_2_ER) reflects the balance between DO_2_ and VO_2_, a GDT protocol based on O_2_ER estimation (O_2_ERe) calculated as (SaO_2_-ScvO_2_)/SaO_2_ (where SaO_2_ is arterial oxygen saturation) has been proposed showing a significantly lower number of organ failure postoperatively compared with control group [8].

The major determinants of DO_2_ are CO, hemoglobin (Hb) level and SaO_2_. An inadequate CO may be optimized using fluids as first line therapy and then inotropes. In mechanically ventilated patients, heart-lung interaction is useful to recognize in which portion of the Frank-Starling curve the heart of the patient is working and then if CO is able to rise after fluid administration aimed to increase preload. Several parameters based on mini-invasive monitor systems are available to assess fluid responsiveness such as pulse pressure variation (PPV) and stroke volume variation (SVV) [9]. Optimization of functional parameters allows titration of fluid administration and personalization of therapy for each patients [10]. Thus, several perioperative GDT protocols are based on fluids administration as long as PPV or SVV is above a pre-defined cut-off, aiming to preload maximization.

Although positive effects of perioperative GDT have been clearly demonstrated, currently studies comparing different strategies of hemodynamic optimization are lacking. To date, only one trial investigated the effect of a protocol based on O_2_ERe [8], whereas many studies were based on functional parameters of fluid-responsiveness (PPV or SVV). Thus, we believe useful a trial comparing different strategy of hemodynamic optimization.

## Methods/design

The protocol adheres to the Standard Protocol Items: Recommendations for Interventional Trials (SPIRIT) 2013 guidelines [11].

### Study aim

The aim of the study is to compare a perioperative hemodynamic optimization protocol based on O_2_ERe referred to a protocol based on normalization of dynamic parameters of fluid-responsiveness (PPV or SVV).

### Objectives

The primary objective is to evaluate the difference in developing overall complications during hospital stay between the two group of patients managed with two GDT protocols using a non-inferiority approach.

The secondary outcomes are the difference between the total amount of fluid administered, the total fluid balance, the needs of vasopressor/inotropes, the hospital length of stay and the mortality at day 28.

### Study design and setting

This is a multicenter randomized controlled trial. As both PGDT protocols showed to reduce postoperative complications compared to standard therapy and they are routinely applied for this kind of patients in common clinical practice [8, 12], a non-inferiority trial will be performed as the effect on primary outcome is expected to be maintained in both groups and it does not seem reasonable for ethical reason to deprive a study group of an established strategy for hemodynamic management during perioperative period. The study will be performed in 15 centers in Italy (Additional file 1).

### Subjects and study population

#### Inclusion criteria

- Adult patients (age > 18 years) undergoing to general anesthesia and mechanical ventilation for elective major open abdominal surgery (gastrointestinal, urologic, gynecologic and vascular surgery)
- Expected duration of surgical procedure higher than 120 min
- ASA-PS Classification II-III-IV
- Planned postoperative ICU/HDU admission

#### Exclusion criteria

- Pregnancy
- Cardiac arrhythmia
- Non-correctable arterial curve alterations
- Undergoing palliative surgery
- Denial of consent to participate

### Randomization

After screening for inclusion and exclusion criteria, patients suitable to be enrolled will be allocated with 1:1 ratio to randomly receive one of the two protocol of perioperative hemodynamic optimization. A computer-based system will be used to create a block randomization list (block size of 6). The allocation sequence concealment will be guaranteed by sealed opaque envelopes. Patients and researchers that perform postoperative follow up will be blinded to the allocated protocol.

### General care and procedures

Patients undergoing elective non-cardiac surgery will be screened to participate to the study. They will be enrolled and allocated to one of the two groups if they meet all inclusion criteria and none of exclusion ones. All patients will be undergone to general anesthesia and mechanically ventilated with a tidal volume of 8 ml/kg and a positive end expiratory pressure (PEEP) of 5 cmH2O. FiO_2_ will be set at discretion of attending anesthesiologist to maintain normoxia. An arterial cannula will be used for continuous arterial monitoring. The arterial transducer will be zeroed at the level of the fourth intercostal space in the middle axillar line. The square-wave test will be used to assess the presence of over−/under-dumping on the arterial signal. A CO monitoring system will be used to record hemodynamic parameters. A central venous catheter will be placed after induction of anesthesia. A balanced crystalloid solution at the rate of 1 ml/kg/h will be administered intraoperatively. Any further fluid bolus will be administered according to the protocol in which the patient is allocated. Balanced crystalloid solutions are the only fluids permitted. Packed red blood cells will be transfused to maintain Hb ≥8 g/dl, at discretion of attending anesthesiologist. Mean arterial pressure will be maintained between 60 and 100 mmHg modulating anesthetic drugs on surgical stimulus or using vasopressor/vasodilator drugs, at discretion of attending anesthesiologist.

### Protocol a

**Fig. 1:**
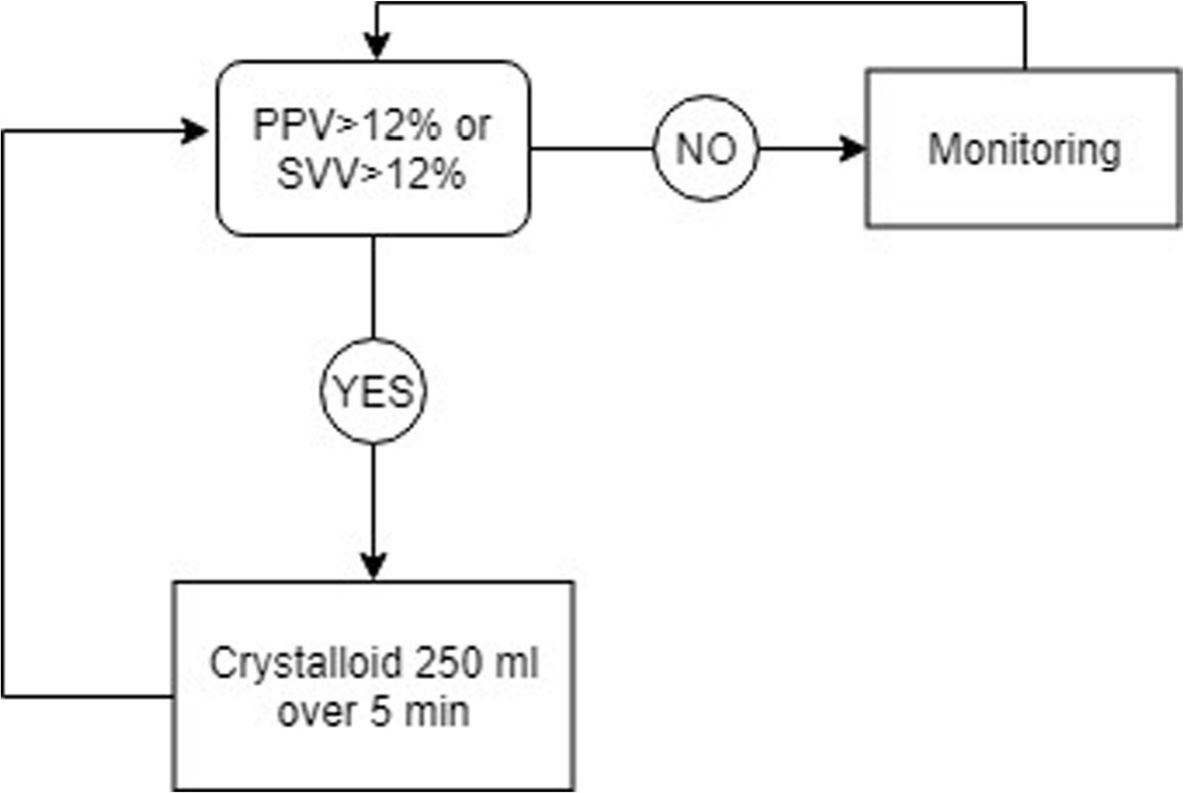
Protocol A: Hemodynamic optimization protocol based on dynamic parameters of fluid responsiveness. PPV: pulse pressure variation; SVV: stroke volume variation

Patients allocated to protocol A will received a GDT protocol based on dynamic parameters of fluid-responsiveness for optimization of fluid status (Fig. 1). Dobutamine will be used with discretion of the attending anesthesiologist as clinically needed. The protocol will be continued postoperatively up to 6 h when dynamic parameters remain applicable (controlled mechanical ventilation, absence of arrythmia, etc.). On the other hand, fluid challenge technique with 250 ml of crystalloid over 5 min will be used for fluid optimization considering 10% increase in CO as positive response.

PPV is calculated as:

$$ PPV\ \left(\%\right)=\frac{\mathrm{PPmax}-\mathrm{PPmin}}{\left(\mathrm{PPmax}+\mathrm{PPmin}\right)/2}X\ 100 $$

where PP is pulse pressure.

SVV is calculated as:

$$ SVV\ \left(\%\right)=\frac{\mathrm{SVmax}-\mathrm{SVmin}}{\left(\mathrm{SVmax}+\mathrm{SVmin}\right)/2}X\ 100 $$

where SV is stroke volume.

### Protocol B

**Fig. 2:**
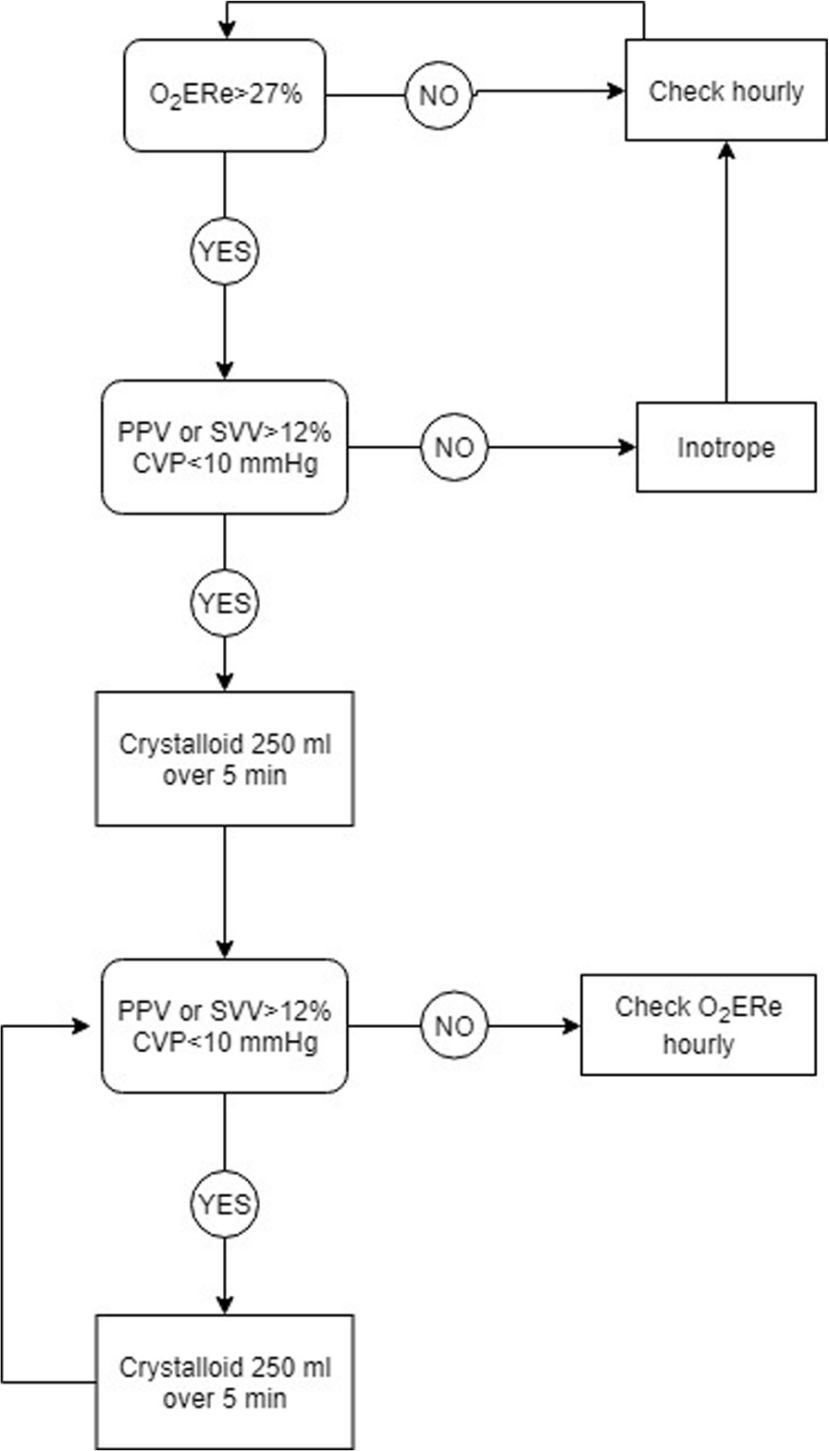
Protocol B: Hemodynamic optimization protocol based on estimated oxygen extraction rate. CVP: central venous pressure; O_2_ERe: oxygen extraction rate estimate; PPV: pulse pressure variation; SVV: stroke volume variation

Patients allocated to protocol B will received a GDT protocol based on O_2_ERe intraoperatively and for the first 6 h postoperatively (Fig. 2).

O_2_ER is estimated at the start of the surgery and then hourly as:

$$ O2 ERe=\frac{SaO2- ScvO2}{SaO2} $$

where SaO_2_ is arterial oxygen saturation and ScvO_2_ is central venous oxygen saturation.

### Data collection

At randomization, demographic and clinical data will be recorded:

- Age, sex, weight, height
- Admission diagnosis
- Comorbidities (e.g. diabetes mellitus, chronic kidney injury, chronic obstructive pulmonary disease, cardiac dysfunction, etc.)

During perioperative period, main clinical parameters will be recorded:

- Ventilation modality and parameters (spontaneous, assisted/controlled, tidal volume, respiratory rate, airway pressures, FiO_2_)
- Anesthetic/analgesic drugs
- Hemodynamic parameters (blood pressure, heart rate, CO, SV, SVR, PPV, SVV)
- Arterial blood gas
- Central venous blood gas
- Fluids (crystalloids and colloids), transfusion and fluid balance
- Inotropic/vasoactive drugs
- Daily SOFA score

Finally, the following outcomes will be recorded:

- Complication during hospital stay (Table 1)
- Hospital length of stay
- Mortality at day 28

**Table 1:** Complications assessed during postoperative period

Acute myocardial ischemia/infarction	Multiorgan failure
Cardiac arrhythmia	Delirium/psychosis
Cardiac/respiratory arrest	Urinary tract infection
Cardiogenic pulmonary edema	Bacteremia
Pulmonary embolism	Surgical site infection
Acute respiratory distress syndrome	Nosocomial pneumonia
Gastrointestinal bleeding	Unknow origin infection
Intestinal infarction	Stroke
Anastomotic leakage	Post-operative hemorrhage
Paralytic ileum	Surgical re-intervention
Acute kidney injury	

Severity of postoperative complications will be defined according to Clavien-Dindo classification [13]. Complication grades I and II will be considered as minor complications while grades III and IV will be considered as major ones.

The Case Report Forms (CRFs) will not bear the participant’s name or other directly identifiable data. The participant’s trial Identification Number (ID) only, will be used for identification.

**Fig. 3:**
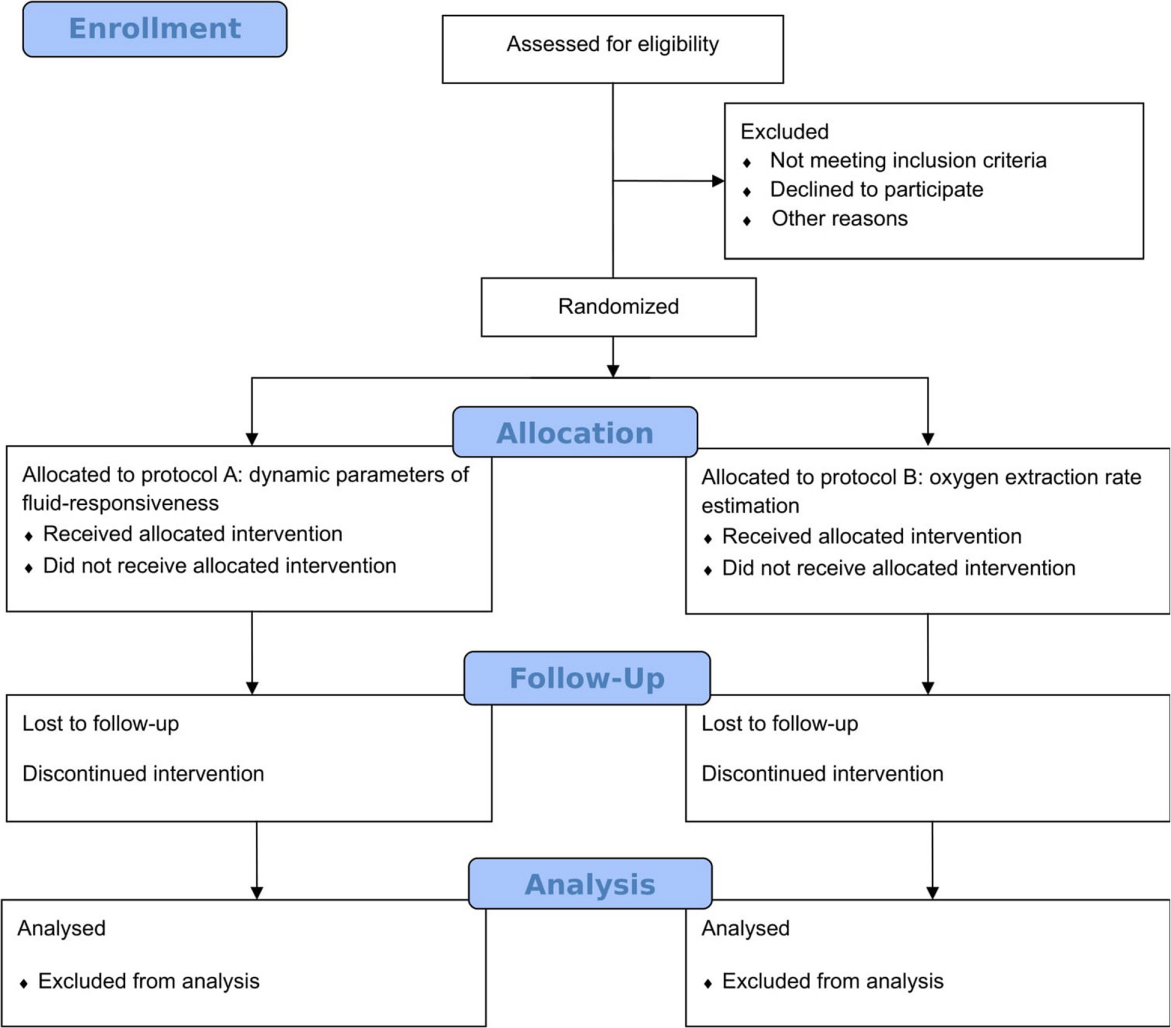
Flow diagram

**Fig. 4:**
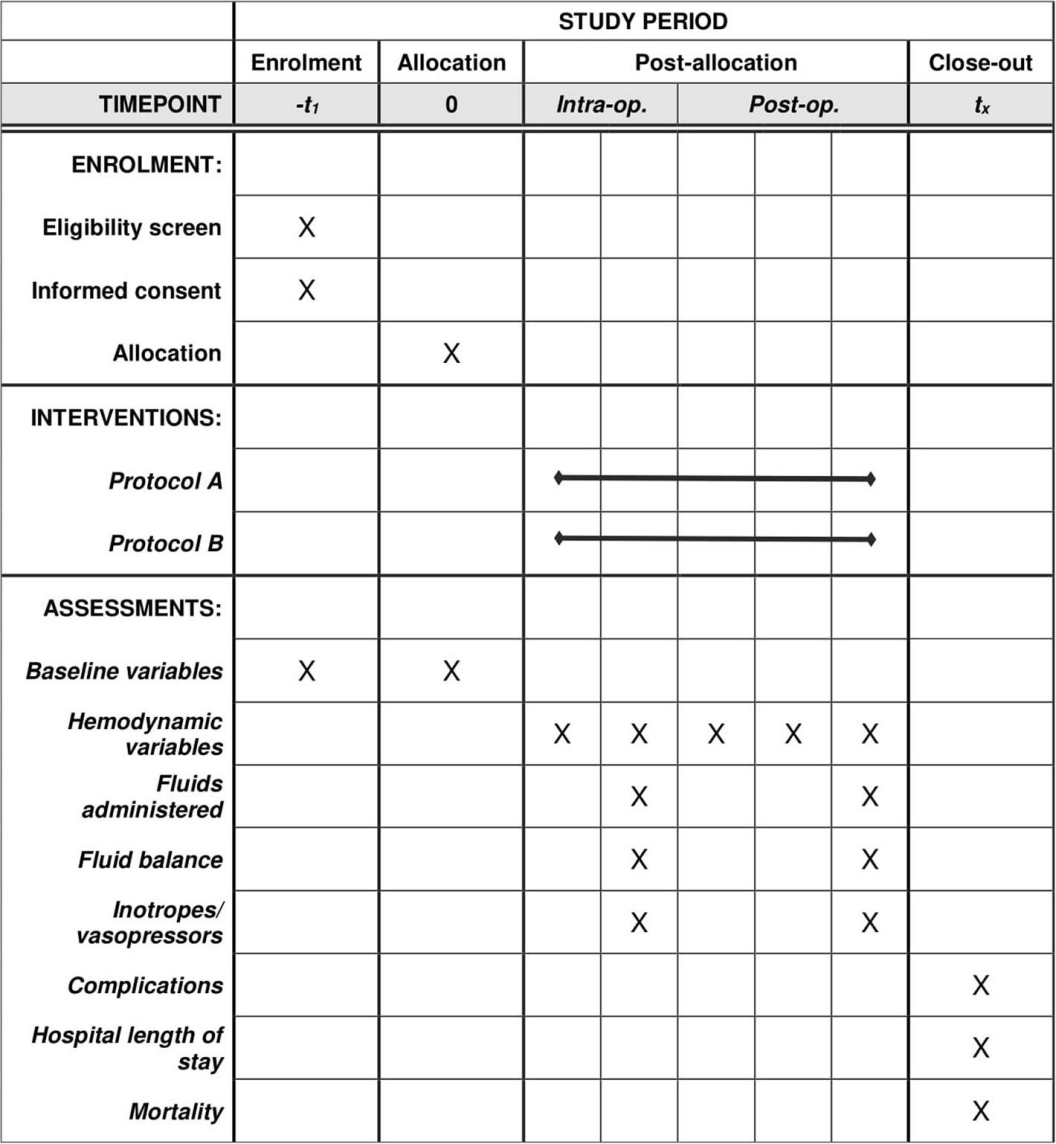
Study timeline

The flow diagram according to CONSORT guidelines [14] is provided as Fig. 3. Figure 4 shows an overview of all outcome measures in accordance with SPIRIT guidelines [11].

### Adverse events

All adverse events (AE) will be recorded in the hospital notes in the first instance. A record of all AEs, whether related or unrelated to the treatment will also be kept in the CRF. If the Investigator suspects that the disease or condition has progressed faster due to the intervention, then he will report this as an unexpected adverse event to the sponsor. Only deaths that are assessed to be caused by the trial intervention will be reported to the Sponsor. This report will be immediate.

Considering the safety profile of the interventions applied in this study (PGDT protocols that showed a benefit to reduce postoperative complication rate without significant adverse effects), we believe that institution of a safety committee is not mandatory.

### Sample size calculation

From literature analysis, we estimate a complication rate for control and experimental group of 28 and 35% respectively [2, 15]. With a non-inferiority margin of 10%, 184 patients (92 for each group) are needed to have a power of 80% and an alfa error of 0.05 [16]. Considering a 10% losing in follow-up, we enroll 200 patients (100 for each group).

### Statistical analysis

Analysis will be performed on intention-to-treat basis.

Data will be checked for normal distribution using Kolmogorov-Smirnov test and presented as mean and standard deviation or as median and interquartile range as appropriate.

The primary end point is the difference of postoperative complication rate between the two group of patients. It will be evaluated using Fisher’s exact test or Pearson’s Chi square test as appropriate. Non-inferiority of experimental protocol respect to control group will be confirmed if the difference (including 95% confidence interval) will be lower than non-inferiority margin established at 10%. The 10% margin was chosen because it represents a two-thirds proportion of lower expected beneficial effect of PGDT (considering higher 95%CI for RR of 0.85) to reduce postoperative complications in comparison with conventional fluid therapy (RR 0.76 [CI95% 0.68–0.85]) [17].

Student t test or Mann–Whitney U test will be used to analyzed secondary outcomes as appropriate. Mortality between groups will be analyzed using the Kaplan-Meier method. The Log Rank test will be used to evaluate the statistical significance. Cox regression model using forward selection method for entering explanatory variables will be used to perform multivariate analysis. The age, type of surgery, ASA-PS classification and comorbidities will be considered as variables. A *p* value of F-test < 0.05 will be considered for variable inclusion. The amount of multicollinearity in the model will be estimated by the variance inflation factor (VIF). A VIF higher than 4 will be considered a sign of multicollinearity. Predicted R^2^ will be used to assess overfitting.

An interim analysis will be performed by independent statistician when 50% of information will be available (100 patients recruited). To monitor for harm or futility, the one-sided *p* value is calculated for testing the hypothesis HR = 1 versus the alternative HR > 1 (meaning the experimental treatment is doing worse than the standard treatment). If the *p* value is < 0.0394 at a monitoring time, then the trial would stop with the conclusion that non-inferiority cannot be claimed [18, 19]. Stopping rule for efficacy has not been defined [20].

For each statistical test, a *p* value < 0.05 is considered statistically significant.

## Discussion

The role of perioperative hemodynamic optimization has been clearly shown by literature and the current spreading of mini-invasive monitoring systems able to estimate CO permits an easier application of these protocols than occurring in the past when only invasive techniques (e.g. pulmonary artery catheter) were available. As a predefined target of CO or DO_2_ seems to be not adequate for every patient, a personalized therapy is likely more appropriate. Following this concept, dynamic parameters of fluid-responsiveness (PPV and SVV) allow to titrate fluid administration aiming an increase of CO as a consequence of preload optimization but avoiding fluid overload. This approach has the advantage of personalized fluid therapy, but it does not consider if CO is adequate or not because preload optimization occurs independently from parameters of tissue perfusion and oxygen consumption. A protocol based on O_2_ER considers this second important aspect. An O_2_ER higher than a predefined cutoff is reflecting an inadequate DO_2_ to tissue and consequently an inadequate CO. This condition, but not fluid-responsiveness per se, is the trigger to start hemodynamic optimization. Moreover, in this contest we believe that CVP may be useful to limit fluid administration independently from PPV or SVV value. In fact, CVP depends on several conditions including preload status but also on cardiac function and ventricular compliance. Thus, this trial aims to demonstrate that this latest strategy of perioperative hemodynamic optimization is not inferior to the former in term of postoperative complications, but it may be responsible for a lower fluid administration. We decided to conduct a non-inferiority study because PGDT is routinely applied in this kind of patients due to the demonstrated benefits on outcome. Thus, small difference in reduction of postoperative complication rate between two different PDGT protocols seems reasonable.

Methodologically, the main limitation of this trial is the inability to blindly administer the interventions. However, this point is not clinically resolvable in this research field. Moreover, if participation of several centers may increase enrollment rate and may potentially extend the applicability of results, on the other hands too few patients per center may increases heterogeneity.

## Trial status

Protocol version 2.0 of 26.06.2019. At the date of manuscript submission, the patients’ recruitment process is ongoing. It is expected that the recruitment will be complete within December 2021. The results of this study have not already been published or submitted to any journal.

## Supplementary information

**Additional file 1.** Participating centers list.

## Data Availability

The datasets used and/or analyzed during the current study are available from the corresponding author on reasonable request.

## Abbreviations

ASA-PS: American Society of Anesthesiologists Physical Status
CO: Cardiac output
DO_2_: Oxygen delivery
GDT: Goal directed therapy
Hb: Hemoglobin
HDU: High dependency unit
ICU: Intensive care unit
O_2_ER: Oxygen extraction rate
O_2_ERe: Oxygen extraction rate estimate
PEEP: Positive end expiratory pressure
PP: Pulse pressure
PPV: Pulse pressure variation
SaO_2_: Arterial oxygen saturation
ScvO_2_: Central venous oxygen saturation
SOFA: Sequential organ failure assessment
SvO_2_: Mixed venous oxygen saturation
SV: Stroke volume
SVV: Stroke volume variation
VO_2_: Oxygen consumption
