# Supplementary material for: Estimated oxygen extraction versus dynamic parameters of fluid-responsiveness for perioperative hemodynamic optimization of patients undergoing non-cardiac surgery: a non-inferiority randomized controlled trial
Source: BMC Anesthesiol. 2020 Apr 18;20:87. doi: 10.1186/s12871-020-01011-z (PMC7165409; doi:10.1186/s12871-020-01011-z)
Supplement: Supplementary file 1 — Additional file 1. Participating centers list. [file 12871_2020_1011_MOESM1_ESM.pdf]

Participating centers list:

1. Anesthesia and Intensive Care Unit, Azienda Ospedaliero Universitaria Ospedali Riuniti, Ancona, Italy
2. Anesthesia and Post-operative Intensive Care Unit, Azienda Ospedaliero Universitaria Ospedali Riuniti, Ancona, Italy
3. Pediatric Anesthesia and Intensive Care Unit, Azienda Ospedaliero Universitaria Ospedali Riuniti, Ancona, Italy
4. Anesthesia and Intensive Care Unit, Azienda Ospedaliera Marche Nord, Pesaro, Italy
5. Anesthesia and Intensive Care Unit, ASUR Marche, Zona territoriale n. 1, Urbino, Italy
6. Anesthesia and Intensive Care Unit, ASUR Marche, Zona territoriale n. 2, Jesi, Italy
7. Anesthesia and Intensive Care Unit, ASUR Marche, Zona territoriale n. 2, Senigallia, Italy
8. Anesthesia and Intensive Care Unit, ASUR Marche, Zona territoriale n. 3, Camerino, Italy
9. Anesthesia and Intensive Care Unit, ASUR Marche, Zona territoriale n. 3, Civitanova Marche, Italy
10. Anesthesia and Intensive Care Unit, ASUR Marche, Zona territoriale n. 3, Macerata, Italy
11. Anesthesia and Intensive Care Unit, ASUR Marche, Zona territoriale n. 4, Fermo, Italy
12. Anesthesia and Intensive Care Unit, ASUR Marche, Zona territoriale n. 5, Ascoli Piceno, Italy
13. Anesthesia and Intensive Care Unit, ASUR Marche, Zona territoriale n. 5, San Benedetto del Tronto, Italy
14. Anesthesia and Intensive Care Unit, IRCCS-INRCA, Ancona, Italy
15. Anesthesia and Intensive Care Unit, IRCCS-INRCA, Osimo, Italy
